# Longitudinal trajectories of walking speed and risk of incident hip fracture in osteoporosis: a group-based trajectory modeling analysis from HRS, ELSA and SHARE

**DOI:** 10.3389/fpubh.2026.1857692

**Published:** 2026-07-02

**Authors:** Hankai Qiu, Minxia Wang, Yi Chen, Rongsheng Chen

**Affiliations:** 1 Fujian Medical University, Fuzhou, Fujian, China; 2 Department of Spinal Surgery, The First Affiliated Hospital of Fujian Medical University, Fuzhou, China; 3 National Regional Medical Center, Binhai the First Affiliated Hospital, Fujian Medical University, Fuzhou, China

**Keywords:** aging, group-based trajectory modeling, hip fracture, osteoporosis, trajectory, walking speed

## Abstract

**Background:**

Walking speed is an objective indicator of physical function and may predict adverse outcomes. However, its longitudinal patterns and association with hip fracture risk in osteoporosis remain unclear.

**Methods:**

Data from HRS, ELSA, and SHARE were analyzed. Participants aged ≥50 years with osteoporosis and no baseline hip fracture were included. Walking speed trajectories were identified using Group-Based Trajectory Modeling. Cox models assessed associations with incident hip fracture, with validation via competing risk and discrete-time models. Dose–response and mediation analyses were also conducted.

**Results:**

A total of 14,139 participants were included. During follow-up, 590 hip fractures occurred. Higher baseline walking speed was associated with increased fracture risk in HRS and ELSA but not SHARE. Four trajectory groups were identified, with risk increasing across groups. The high-increasing trajectory showed the greatest risk (HRS: HR = 2.56; ELSA: HR = 4.54). Results were robust across analyses. A linear relationship was observed, and depression and grip strength partially mediated the association.

**Conclusions:**

Both baseline and longitudinal increases in walking speed were associated with higher hip fracture risk in osteoporosis, highlighting the importance of dynamic functional assessment.

## 1 Introduction

Osteoporosis represents a major public health challenge in aging populations. It is characterized by progressive bone loss and microarchitectural deterioration, which increase skeletal fragility and susceptibility to fractures, particularly hip fractures.Global epidemiological evidence indicates that hip fractures remain highly prevalent among older adults. Although incidence rates of hip fracture have stabilized in some regions due to improved preventive strategies, the overall burden of hip fracture remains substantial in the context of population aging (1, 2).

Among individuals with osteoporosis, hip fractures occur at disproportionately high rates and are associated with poor prognosis. The 1-year mortality rate typically exceeds 20%−30%, with pneumonia, thromboembolism, and cardiovascular events representing the leading causes of death (1, 3). Survivors frequently experience significant functional decline, including impaired mobility and reduced ability to perform activities of daily living, often resulting in permanent disability and increased reliance on long-term institutional care (3, 4). These outcomes not only reduce quality of life but also impose considerable socioeconomic burdens due to prolonged hospitalization and rehabilitation requirements (4, 5).

Walking speed, commonly assessed over short distances, is a reliable and non-invasive biomarker of dynamic physical function in older adults. It reflects the integrated performance of multiple physiological systems, including neuromuscular coordination, lower-extremity muscle strength, balance, and cardiovascular reserve (6, 7). A gradual decline in walking speed indicates deterioration in physical function, which can limit participation in physical and social activities. This limitation may initiate a vicious cycle characterized by reduced fitness, muscle loss, and social isolation (8, 9). Substantial evidence has shown that reduced walking speed and its long-term trajectories are associated with increased risks of various chronic conditions, including cardiovascular disease, neurodegenerative disorders, frailty, and all-cause mortality (10–12). Importantly, most previous studies have consistently demonstrated that slower walking speed is associated with adverse health outcomes, including frailty, falls, disability, and mortality in older adults. Therefore, walking speed is generally regarded as a marker of physiological reserve and healthy aging. However, whether this conventional relationship remains applicable in high-risk populations with osteoporosis remains uncertain, particularly because fracture occurrence may depend not only on physical function itself but also on fall exposure and skeletal fragility.

However, the relationships between baseline walking speed, its longitudinal trajectories, and the incidence of hip fracture among individuals with osteoporosis remain unclear. Although previous cross-sectional and short-term studies have identified slow gait as a risk factor for falls and fractures (13), longitudinal analyses focusing on trajectory patterns in high-risk osteoporotic populations are limited, leaving a critical gap in the literature. In addition, emerging evidence suggests that changes in walking speed and its trajectories may predict incident depression in later life. Potential mechanisms include systemic inflammation and oxidative stress, which may impair both motor function and emotional regulation through neurovascular and musculoskeletal pathways (8, 14, 15).

**Figure 1:**
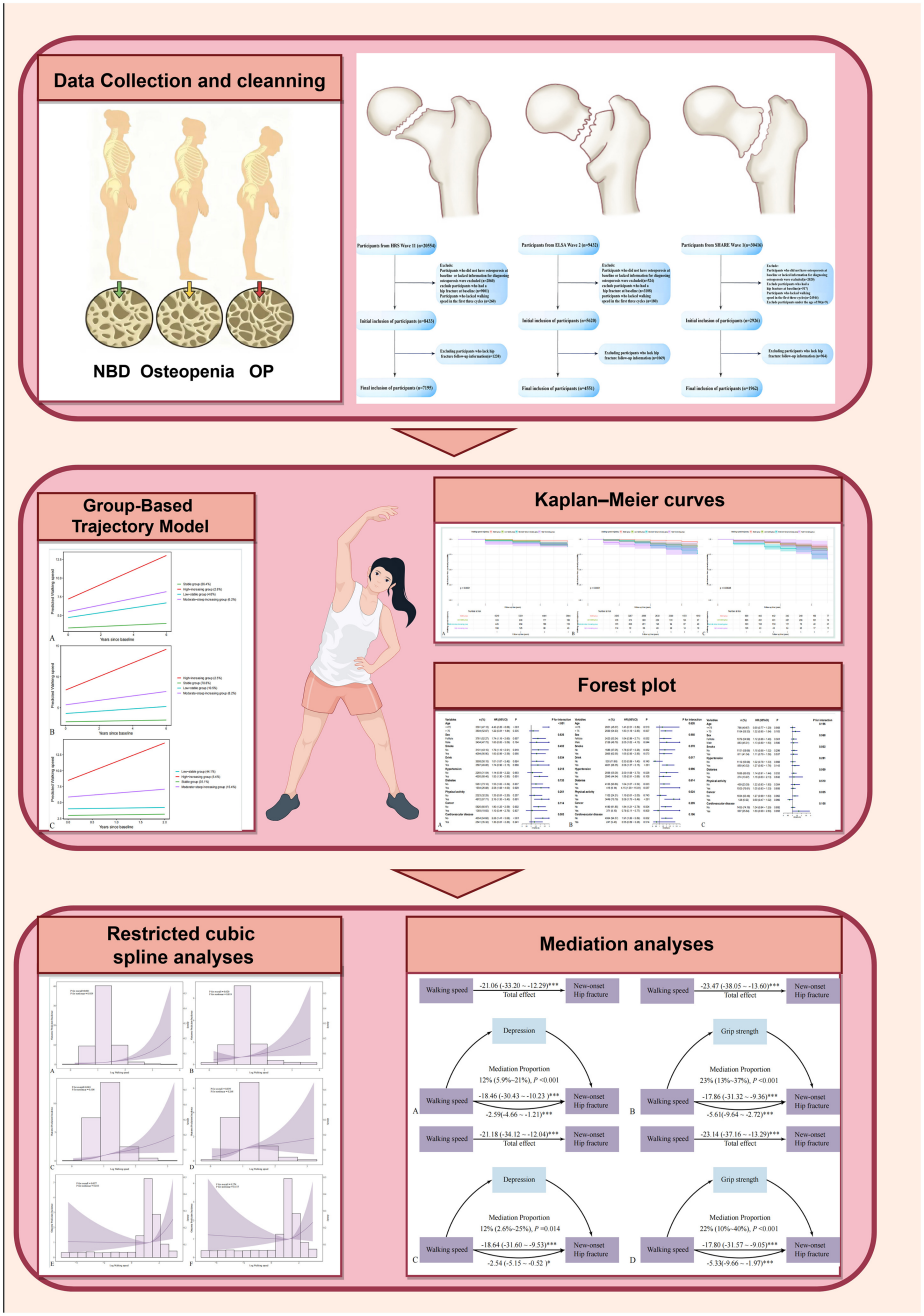
Graphical abstract.

To address these gaps, the present study utilized longitudinal data from two large, harmonized population-based cohorts—the Health and Retirement Study (HRS), the English Longitudinal Study of Aging (ELSA) and the Survey of Health, Aging and Retirement in Europe (SHARE)—to comprehensively examine the associations between walking speed, its trajectory patterns, and the risk of incident hip fracture among individuals with osteoporosis. The overall study design and analytical workflow are summarized in (Figure 1).

## 2 Materials and methods

### 2.1 Study population

The ELSA, conducted by a consortium led by University College London and the National Center for Social Research, is designed to monitor the health, economic, and social conditions of adults aged ≥50 years living in private households in England. The HRS, conducted by the University of Michigan with support from the National Institute on Aging, examines the dynamic interplay between health, economic, and social factors among adults aged ≥51 years in the non-institutionalized population of the United States. The SHARE, coordinated by the SHARE-ERIC consortium and the Munich Center for the Economics of Aging at the Max Planck Institute for Social Law and Social Policy, is a cross-national longitudinal study that collects harmonized data on health, socioeconomic status, and social and family networks among community-dwelling adults aged ≥50 years across multiple European countries.

**Figure 2:**
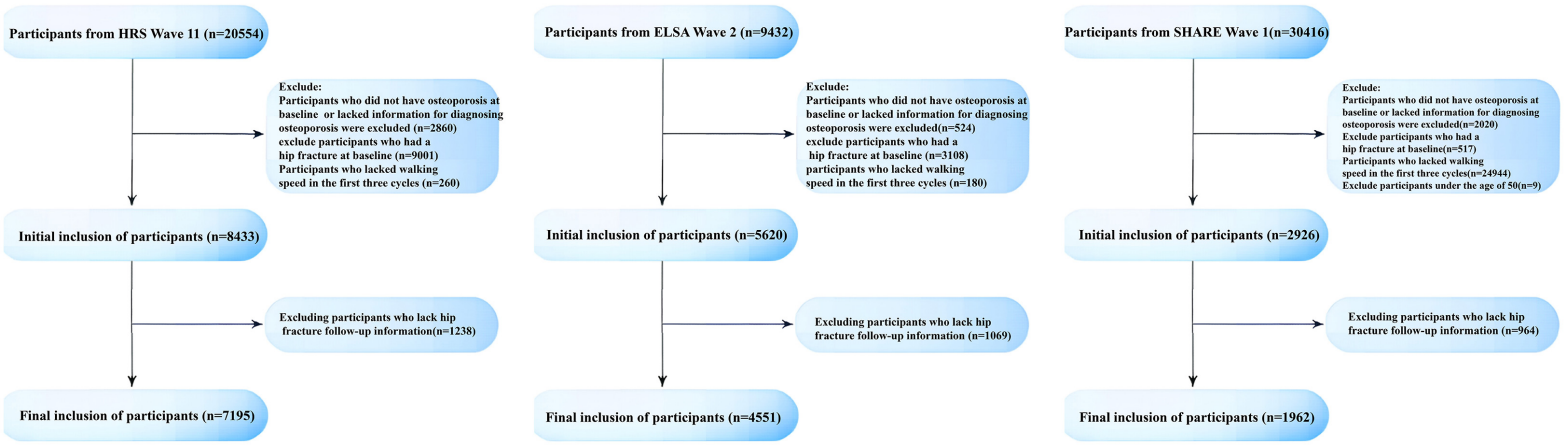
Flowchart of participant selection.

This longitudinal cohort study was based on data from HRS, ELSA, and SHARE, all of which are nationally representative, ongoing studies of community-dwelling older adults. Standardized interviews and physical examinations are conducted biennially to obtain detailed information on sociodemographic characteristics, health status, functional capacity, and health-related behaviors. The participant selection process is illustrated in (Figure 2).

In the HRS database, 20,554 participants were initially identified. After applying predefined exclusion criteria, 13,359 individuals were excluded: (1) absence of osteoporosis or missing diagnosis during the first three waves (*n* = 2,860); (2) presence of hip fracture or missing diagnosis during the first three waves (*n* = 9,001); (3) missing walking speed data during the first three waves (*n* = 260); and (4) lack of follow-up information on hip fracture (*n* = 1,238). A total of 7,195 participants were ultimately included (Figure 2).

In the ELSA database, 9,432 participants were initially included, of whom 4,881 were excluded: (1) absence of osteoporosis or missing diagnosis during the first three waves (*n* = 524); (2) presence of hip fracture or missing diagnosis during the first three waves (*n* = 3,108); (3) missing walking speed data during the first three waves (*n* = 180); and (4) lack of follow-up information on hip fracture (*n* = 1,069). The final analytical sample comprised 4,982 participants (Figure 2).

In the SHARE database, 30,416 participants were initially identified. A total of 28,454 individuals were excluded: (1) absence of osteoporosis or missing diagnosis during the first three waves (*n* = 2,020); (2) presence of hip fracture or missing diagnosis during the first three waves (*n* = 517); (3) missing walking speed data during the first three waves (*n* = 24,944); (4) age < 50 years (*n* = 9); and (5) lack of follow-up information on hip fracture (*n* = 964). Ultimately, 1,962 participants were included in the analysis (Figure 2).

### 2.2 Outcome

#### 2.2.1 Assessment of walking speed and construction of trajectories

Walking speed was assessed using standardized physical performance tests across HRS, ELSA, and SHARE. In all cohorts, participants were instructed to complete an 8-foot walk at their usual pace, and walking speed was calculated as distance divided by completion time and expressed in meters per second (m/s). Two measurements were obtained, and the average value was used for analysis to improve measurement reliability.

To characterize long-term dynamic patterns, trajectory analyses were conducted using walking speed data from three consecutive waves. Only participants with valid measurements across all three waves were included. Time was defined as years since baseline, with approximately 2-year intervals between waves, to capture longitudinal changes in physical function. Group-Based Trajectory Modeling (GBTM) was applied to identify latent longitudinal patterns of walking speed across the three assessment waves, with walking speed repeatedly measured over time. Models with varying numbers of trajectory groups were fitted. After selecting the optimal model, each participant was assigned to a trajectory group based on the maximum posterior probability. These groups were subsequently treated as categorical variables in association analyses.

#### 2.2.2 Assessment of osteoporosis and incident hip fracture

Osteoporosis was defined based on self-reported physician diagnosis in all cohorts, and incident hip fracture was identified according to the first self-reported physician diagnosis during follow-up using standardized questionnaires. Because standardized imaging data and medical record verification were not consistently available across the three cohorts, self-reported diagnoses were used to ensure harmonized definitions across datasets.

To ensure appropriate temporal ordering, all participants were free of hip fracture during the trajectory modeling period (the first three waves). Because HRS, ELSA, and SHARE collected outcome information at biennial follow-up interviews rather than recording exact event dates, the timing of incident hip fracture was defined according to the follow-up wave in which the fracture was first reported. Accordingly, follow-up time was calculated from the end of the trajectory assessment period to the corresponding interview wave, with participants censored at the time of hip fracture report, loss to follow-up, death, or the end of follow-up. This design ensured that walking speed trajectories preceded the outcome, thereby minimizing potential reverse causation. Only participants with osteoporosis were included.

To ensure appropriate temporal ordering, all participants were free of hip fracture during the trajectory modeling period (the first three waves). Because HRS, ELSA, and SHARE collected outcome information at biennial follow-up interviews rather than recording exact event dates, the timing of incident hip fracture was defined according to the follow-up wave in which the fracture was first reported. Accordingly, follow-up time was calculated from the end of the trajectory assessment period to the corresponding interview wave, with participants censored at the time of hip fracture report, loss to follow-up, death, or the end of follow-up. This design ensured that walking speed trajectories preceded the outcome, thereby minimizing potential reverse causation. Only participants with osteoporosis were included.

### 2.3 Covariates

Sociodemographic and lifestyle variables were included as covariates, including age, sex, race, education, wealth, body mass index (BMI), alcohol consumption, smoking status, hypertension, diabetes, cancer, physical activity, and cardiovascular disease (CVD). CVD was defined as a self-reported physician diagnosis of major cardiovascular conditions, including stroke, angina, heart failure, and related cardiovascular disorders. Hypertension was analyzed separately as an independent cardiometabolic risk factor. Smoking status was categorized as never or current smoker, and alcohol consumption as non-drinker or current drinker. BMI was calculated as weight (kg) divided by height squared (m^2^). Diabetes was defined based on self-reported physician diagnosis (“Doctor told you have diabetes”). In the SHARE cohort, alcohol consumption data were unavailable for the selected waves; therefore, this variable was not included in SHARE-specific analyses.

### 2.4 Statistical analyses

Continuous variables were expressed as mean ± standard deviation (SD), and categorical variables were presented as counts and percentages. Group differences were assessed using the chi-square test or Student's *t*-test, as appropriate. To evaluate potential selection bias, baseline characteristics between included and excluded participants in the SHARE cohort were additionally compared.

GBTM, based on finite mixture models, was used to identify distinct patterns of walking speed change. This approach applies maximum likelihood estimation to classify individuals with similar developmental trajectories into latent subgroups. Trajectory modeling was conducted separately within each cohort using walking speed data from the first three waves, with time defined as years since baseline. Models with one to four trajectory groups and different polynomial functional forms were systematically evaluated. The optimal model was determined based on multiple criteria, including the Bayesian Information Criterion (BIC), Akaike Information Criterion (AIC), log-likelihood values, mean posterior probability, group membership proportions, and clinical interpretability of the trajectories. Model selection criteria included lower BIC values, a minimum group size >5%, and a mean posterior probability ≥0.70. Polynomial orders were selected according to model fit and trajectory stability. The final four-group model was selected because it demonstrated the best overall balance between statistical fit, classification accuracy, trajectory interpretability, and subgroup stability. Each participant was assigned to a trajectory group according to the maximum posterior probability. Detailed model selection results, including competing trajectory solutions, posterior probabilities, and polynomial specifications, are provided in (Supplementary Tables 1–3).

Multivariable Cox proportional hazards regression models were used to examine the associations between walking speed, its trajectories, and the risk of incident hip fracture. Time-to-event was defined as the interval from the end of the initial three-wave assessment period to the first occurrence of hip fracture, censoring, or the end of follow-up. Hazard ratios (HRs) and 95% confidence intervals (CIs) were estimated. Covariates were entered sequentially to evaluate the robustness of the associations. Model 2 adjusted for major demographic and anthropometric factors. Model 3 further included cardiometabolic and lifestyle-related variables, including hypertension, alcohol consumption, smoking status, and diabetes, because these factors may influence both walking speed and fracture risk. Model 4 additionally adjusted for cancer, CVD, and physical activity to further account for chronic disease burden and behavioral factors related to mobility, frailty, and skeletal health. The proportional hazards assumption was evaluated using Schoenfeld residuals, and no substantial violations were detected.

Given that death may preclude the occurrence of hip fracture, competing risk regression based on the Fine–Gray subdistribution hazards model was performed, with death treated as a competing event. Subdistribution hazard ratios (sHRs) and 95% CIs were reported. To further assess robustness under an alternative time framework, discrete-time logistic regression models were fitted using follow-up waves as the time unit. Data were restructured into a person-period format, with each participant contributing one observation per wave until incident hip fracture or censoring.

Restricted cubic spline (RCS) analyses were conducted to assess potential non-linear relationships between walking speed and incident hip fracture. Walking speed was log-transformed in each cohort to approximate normality and to facilitate comparison of effect estimates across cohorts. All analyses were performed using IBM SPSS Statistics (version 24.0) and R software (version 4.3.0). A two-sided *P*-value < 0.05 was considered statistically significant.

As an additional sensitivity analysis, fall history was further included as an additional covariate in the fully adjusted Cox regression models to evaluate the potential influence of fall-related confounding on the observed associations.

## 3 Results

### 3.1 Baseline characteristics stratified by new-onset hip fracture

Tables summarize the baseline characteristics of osteoporotic participants, stratified by the incidence of new-onset hip fractures, across the HRS, ELSA, and SHARE databases. The HRS cohort included 7,195 osteoporotic participants who were free of hip fractures during the preceding three waves. This cohort had a mean age of 75.00 years (SD = 6.81) and comprised 3,761 females (52.27%) and 3,434 males (47.73%). Over a median follow-up of 8 years, 232 participants developed a new-onset hip fracture (mean age 78.16 years, SD = 7.41). Compared to the non-fracture group, individuals who sustained a fracture were significantly older (mean 78.16 vs. 74.89 years, *P* < 0.001), predominantly female (65.95% vs. 34.05%, *P* < 0.001), and from lower-income households (mean 436,739.68 vs. 516,565.57, *P* = 0.009). Additionally, incident fractures were associated with a lower BMI (mean 26.25 vs. 28.25, *P* < 0.001), reduced muscle strength (mean 26.14 vs. 29.44, *P* < 0.001), and a faster walking speed (mean 4.25 vs. 3.64, *P* < 0.001) (Table 1).

**Table 1:** Baseline characteristics of osteoporotic participants stratified by incident hip fracture in the HRS cohort.

Characteristic	*N*	Overall *N* = 7,195	Normal *N* = 6,963	New-onset Hip fracture *N* = 232	*P*-value
Sex^b^, *n* (%)					
Female	7,195	3,761 (52.27%)	3,608 (51.82%)	153 (65.95%)	< 0.001
Male		3,434 (47.73%)	3,355 (48.18%)	79 (34.05%)	
Age (year)^a^, Mean ± SD	7,195	75.00 ± 6.81	74.89 ± 6.77	78.16 ± 7.41	< 0.001
Race^b^, *n* (%)					
Non-Hispanic white	7,195	5,306 (73.75%)	5,130 (73.68%)	176 (75.86%)	0.845
Non-Hispanic Blacks		1,107 (15.39%)	1,074 (15.42%)	33 (14.22%)	
Hispanic		628 (8.73%)	608 (8.73%)	20 (8.62%)	
Other Race		154 (2.14%)	151 (2.17%)	3 (1.29%)	
Education^b^, *n* (%)					
High school or less	7,195	1,637 (22.75%)	1,568 (22.52%)	69 (29.74%)	0.033
Some college		3,938 (54.73%)	3,820 (54.86%)	118 (50.86%)	
College graduate or above		1,620 (22.52%)	1,575 (22.62%)	45 (19.40%)	
Wealth ^a^, Mean ±	7,195	513,991.61 ± 1,130,637.39	516,565.57 ± 1,130,261.75	436,739.68 ± 1,141,611.72	0.009
BMI ^a^, Mean ±	7,195	28.19 ± 5.58	28.25 ± 5.56	26.25 ± 5.92	< 0.001
Hypertension					
No	7,195	2,269 (31.54%)	2,199 (31.58%)	70 (30.17%)	0.650
Yes		4,926 (68.46%)	4,764 (68.42%)	162 (69.83%)	
Diabetes					
No	7,195	5,261 (73.12%)	5,092 (73.13%)	169 (72.84%)	0.923
Yes		1,934 (26.88%)	1,871 (26.87%)	63 (27.16%)	
Smoking status^b^, *n* (%)					
No	7,195	3,101 (43.10%)	2,997 (43.04%)	104 (44.83%)	0.589
Yes		4,094 (56.90%)	3,966 (56.96%)	128 (55.17%)	
Drinking status^b^, *n* (%)					
No	7,195	3,608 (50.15%)	3,456 (49.63%)	152 (65.52%)	< 0.001
Yes		3,587 (49.85%)	3,507 (50.37%)	80 (34.48%)	
Cancer					
No	7,195	5,826 (80.97%)	5,637 (80.96%)	189 (81.47%)	0.846
Yes		1,369 (19.03%)	1,326 (19.04%)	43 (18.53%)	
Physical activity ^b^, *n* (%)					
No	7,195	2,323 (32.29%)	2,220 (31.88%)	103 (44.40%)	< 0.001
Yes		4,872 (67.71%)	4,743 (68.12%)	129 (55.60%)	
Cardiovascular disease					
No	7,195	4,654 (64.68%)	4,520 (64.91%)	134 (57.76%)	0.025
Yes		2,541 (35.32%)	2,443 (35.09%)	98 (42.24%)	
Grip strength ^a^, *n* (%)	7,195	29.34 ± 9.85	29.44 ± 9.84	26.14 ± 9.44	< 0.001
Walking speed ^a^, *n* (%)	7,195	3.66 ± 1.87	3.64 ± 1.82	4.25 ± 3.08	< 0.001
Log Walking speed ^a^, *n* (%)	7,195	1.22 ± 0.36	1.22 ± 0.36	1.33 ± 0.42	< 0.001
**Walking speed ^b^, *n* (%)**					
Stable group	7,195	6,219 (86.44%)	6,052 (86.92%)	167 (71.98%)	< 0.001
Low–stable group		333 (4.63%)	313 (4.50%)	20 (8.62%)	
Moderate–steep increasing group		445 (6.18%)	416 (5.97%)	29 (12.50%)	
High–increasing group		198 (2.75%)	182 (2.61%)	16 (6.90%)	

^a^Student*t*-test; ^*b*^Chi-square test; SD, standard deviation.

The ELSA cohort consisted of 4,551 osteoporotic participants meeting the same fracture-free baseline criteria. The mean age was 70.67 years (SD = 7.61), with 2,423 females (53.24%) and 2,128 males (46.76%). During a median 10-year follow-up, 194 participants sustained a new-onset hip fracture (mean age 74.03 years, SD = 7.80). A higher fracture incidence was similarly observed in older participants (mean 74.03 vs. 70.52, *P* < 0.001), females (61.86% vs. 38.14%, *P* = 0.014), and individuals exhibiting a lower BMI (mean 27.25 vs. 27.88, *P* = 0.037) or weaker muscle strength (51.15 vs. 56.60, *P* < 0.001). A faster walking speed was also positively associated with fracture incidence in this cohort (mean 3.46 vs. 3.24, *P* = 0.004) (Table 2).

**Table 2:** Baseline characteristics of osteoporotic participants stratified by incident hip fracture in the ELSA cohort.

Characteristic	*N*	Overall *N* = 4,551	Normal *N* = 4,357	New-onset Hip fracture *N* = 194	*P*-value
Sex^b^, *n* (%)					
Female	4,551	2,423 (53.24%)	2,303 (52.86%)	120 (61.86%)	0.014
Male		2,128 (46.76%)	2,054 (47.14%)	74 (38.14%)	
Age (year)^a^, Mean ± SD	4,551	70.67 ± 7.61	70.52 ± 7.57	74.03 ± 7.80	< 0.001
Race^b^, *n* (%)					
No white	4,551	76 (1.67%)	76 (1.74%)	0 (0.00%)	0.077
White		4,475 (98.33%)	4,281 (98.26%)	194 (100.00%)	
Education^b^, *n* (%)					
Before high school	4,551	2,422 (53.22%)	2,319 (53.22%)	103 (53.09%)	0.051
High school		750 (16.48%)	720 (16.53%)	30 (15.46%)	
Junior college		835 (18.35%)	788 (18.09%)	47 (24.23%)	
College graduate or above		544 (11.95%)	530 (12.16%)	14 (7.22%)	
BMI^a^, Mean ± SD	4,551	27.86 ± 4.64	27.88 ± 4.63	27.25 ± 4.84	0.037
Wealth ^a^, Mean ± SD	4,551	262,888.27 ± 340,264.46	263,290.17 ± 341,629.41	253,862.29 ± 308,686.42	0.453
Hypertension ^b^, *n* (%)					
No	4,551	2,506 (55.06%)	2,409 (55.29%)	97 (50.00%)	0.147
Yes		2,045 (44.94%)	1,948 (44.71%)	97 (50.00%)	
Diabetes ^b^, *n* (%)					
No	4,551	4,135 (90.86%)	3,955 (90.77%)	180 (92.78%)	0.342
Yes		416 (9.14%)	402 (9.23%)	14 (7.22%)	
Smoking status^b^, *n* (%)					
No	4,551	1,686 (37.05%)	1,616 (37.09%)	70 (36.08%)	0.776
Yes		2,865 (62.95%)	2,741 (62.91%)	124 (63.92%)	
Drinking status^b^, *n* (%)					
No	4,551	530 (11.65%)	509 (11.68%)	21 (10.82%)	0.716
Yes		4,021 (88.35%)	3,848 (88.32%)	173 (89.18%)	
Cancer ^b^, *n* (%)					
No	4,551	4,180 (91.85%)	4,000 (91.81%)	180 (92.78%)	0.626
Yes		371 (8.15%)	357 (8.19%)	14 (7.22%)	
Physical activity^b^, *n* (%)					
No	4,551	1,102 (24.21%)	1,037 (23.80%)	65 (33.51%)	0.002
Yes		3,449 (75.79%)	3,320 (76.20%)	129 (66.49%)	
Cardiovascular disease ^b^, *n* (%)					
No	4,551	4,304 (94.57%)	4,120 (94.56%)	184 (94.85%)	0.864
Yes		247 (5.43%)	237 (5.44%)	10 (5.15%)	
Grip strength^a^, Mean ± SD	4,551	56.37 ± 21.26	56.60 ± 21.25	51.15 ± 21.03	< 0.001
Walking speed^a^, Mean ±SD	4,551	3.25 ± 1.71	3.24 ± 1.70	3.46 ± 1.91	0.004
Log Walking speed ^a^, Mean ± SD	4,551	1.10 ± 0.37	1.09 ± 0.37	1.16 ± 0.37	0.004
Walking speed^b^, *n* (%)					
Stable group	4,551	3,585 (78.77%)	3,464 (79.50%)	121 (62.37%)	< 0.001
Low–stable group		479 (10.53%)	448 (10.28%)	31 (15.98%)	
Moderate–steep increasing group		373 (8.20%)	342 (7.85%)	31 (15.98%)	
High–increasing group		114 (2.50%)	103 (2.36%)	11 (5.67%)	

^a^ Student *t*-test; ^b^ Chi-square test; SD, standard deviation.

In the SHARE database, 1,962 eligible participants were evaluated. The mean age was 77.60 years (SD = 7.62), including 1,079 females (54.99%) and 883 males (45.01%). Over a median 6-year follow-up, 164 new-onset hip fractures were recorded (mean age 78.01 years, SD = 6.66). Increased fracture susceptibility was again noted among older participants (mean 78.01 vs. 77.57, *P* = 0.618), females (64.63% vs. 35.37%, *P* = 0.010), and those with faster walking speeds (mean 4.59 vs. 4.39, *P* = 0.202) (Table 3). Overall, the distribution of baseline characteristics among osteoporotic participants demonstrated considerable consistency across all three independent cohorts. Comparison analyses demonstrated significant differences between included and excluded participants in the SHARE cohort, particularly with respect to age, cardiovascular comorbidities, smoking status, and body mass index (Supplementary Table 4).

**Table 3:** Baseline characteristics of osteoporotic participants stratified by new-onset hip fracture in the SHARE cohort.

Characteristic	*N*	Overall *N* = 1,962	Normal *N* = 1,798	New-onset Hip fracture *N* = 164	*P*-value
Sex^b^, *n* (%)					
Female	1,962	1,079 (54.99%)	973 (54.12%)	106 (64.63%)	0.010
Male		883 (45.01%)	825 (45.88%)	58 (35.37%)	
Age (year)^a^, Mean ± SD	1,962	77.60 ± 7.62	77.57 ± 7.70	78.01 ± 6.66	0.618
Native ^b^, *n* (%)					
No	1,962	31 (1.58%)	28 (1.56%)	3 (1.83%)	0.740
Yes		1,931 (98.42%)	1,770 (98.44%)	161 (98.17%)	
Education^b^, *n* (%)					
Before high school	1,962	1,398 (71.25%)	1,277 (71.02%)	121 (73.78%)	0.067
High school and vocational training		354 (18.04%)	334 (18.58%)	20 (12.20%)	
Higher Education		210 (10.70%)	187 (10.40%)	23 (14.02%)	
Wealth ^a^, Mean ±	1,962	175,088.63 ± 270,213.46	175,075.13 ± 272,301.53	175,236.65 ± 246,930.23	0.943
BMI ^a^, Mean ±	1,962	26.52 ± 4.66	26.53 ± 4.67	26.35 ± 4.56	0.767
Hypertension					
No	1,962	1,112 (56.68%)	1,035 (57.56%)	77 (46.95%)	0.009
Yes		850 (43.32%)	763 (42.44%)	87 (53.05%)	
Diabetes					
No	1,962	1,688 (86.03%)	1,549 (86.15%)	139 (84.76%)	0.622
Yes		274 (13.97%)	249 (13.85%)	25 (15.24%)	
Smoking status^b^, *n* (%)					
No	1,962	1,151 (58.66%)	1,050 (58.40%)	101 (61.59%)	0.428
Yes		811 (41.34%)	748 (41.60%)	63 (38.41%)	
Cancer					
No	1,962	1,834 (93.48%)	1,683 (93.60%)	151 (92.07%)	0.447
Yes		128 (6.52%)	115 (6.40%)	13 (7.93%)	
Physical activity ^b^, *n* (%)					
No	1,962	459 (23.39%)	410 (22.80%)	49 (29.88%)	0.040
Yes		1,503 (76.61%)	1,388 (77.20%)	115 (70.12%)	
Cardiovascular disease ^b^, *n* (%)					
No	1,962	1,455 (74.16%)	1,337 (74.36%)	118 (71.95%)	0.500
Yes		507 (25.84%)	461 (25.64%)	46 (28.05%)	
Walking speed^a^, Mean ± SD	1,962	4.40 ± 2.73	4.39 ± 2.73	4.59 ± 2.63	0.202
Log Walking speed ^a^, Mean ± SD	1,962	1.27 ± 0.85	1.27 ± 0.84	1.31 ± 0.88	0.202
Walking speed^b^, *n* (%)					
Stable group	1,962	689 (35.12%)	637 (35.43%)	52 (31.71%)	
Low–stable group		865 (44.09%)	802 (44.61%)	63 (38.41%)	
Moderate–steep increasing group		303 (15.44%)	263 (14.63%)	40 (24.39%)	
High–increasing group		105 (5.35%)	96 (5.34%)	9 (5.49%)	

^a^Student *t*-test; ^b^chi-square test; SD, standard deviation.

### 3.2 Association between walking speed, trajectories, and fracture risk

Table 4 details the associations between baseline walking speed, temporal trajectories, and fracture risk, analyzed via Cox proportional hazards regression. In crude models, baseline walking speed positively correlated with fracture risk in both the HRS (HR = 2.86, 95% CI: 2.11–3.87, *P* < 0.001) and ELSA cohorts (HR = 2.66, 95% CI: 1.90–3.72, *P* < 0.001). This correlation remained robust following adjustments for potential confounders in Model 4, which included age, sex, race, education, wealth, BMI, drinking and smoking status, hypertension, diabetes, physical activity, cancer, and CVD. Specifically, each one-unit increase in log-transformed walking speed elevated the fracture risk by 62% in the HRS cohort (adjusted HR = 1.62, 95% CI: 1.15–2.28, *P* = 0.006) and 65% in the ELSA cohort (adjusted HR = 1.65, 95% CI: 1.10–2.47, *P* = 0.016).

**Table 4:** Associations between walking speed, its trajectory patterns, and the risk of incident hip fracture among individuals with osteoporosis.

Exposure Variable	Model 1		Model 2		Model 3		Model 4	
	HR (95% CI)	*P*-value	HR (95% CI)	*P*-value	HR (95% CI)	*P*-value	HR (95% CI)	*P-*value
HRS								
Log walking speed	2.86 (2.11–3.87)	< 0.001^***^	2.03 (1.47–2.81)	< 0.001^***^	1.89 (1.35–2.62)	< 0.001^***^	1.62 (1.15–2.28)	0.006^**^
Walking speed trajectory								
Stable group	1.00 (Reference)	–	1.00 (Reference)	–	1.00 (Reference)	–	1.00 (Reference)	–
Low–stable group	2.82 (1.77–4.49)	< 0.001^***^	2.20 (1.36–3.56)	0.001^**^	2.06 (1.27–3.34)	0.003^**^	1.82 (1.12–2.96)	0.015^*^
Moderate–steep increasing group	3.44 (2.32–5.12)	< 0.001^***^	2.51 (1.66–3.79)	< 0.001^***^	2.27 (1.50–3.44)	< 0.001^***^	2.00 (1.32–3.05)	0.001^**^
High–increasing group	4.47 (2.67–7.49)	< 0.001^***^	3.34 (1.97–5.69)	< 0.001^***^	3.10 (1.82–5.27)	< 0.001^***^	2.56 (1.49–4.39)	< 0.001^***^
*P* for trend	11.97 (6.75–21.25)	< 0.001^***^	6.72 (3.62–12.51)	< 0.001^***^	5.68 (3.05–10.61)	< 0.001^***^	4.29 (2.26–8.12)	< 0.001^***^
ELSA								
Log Walking speed	2.66 (1.90–3.72)	< 0.001^***^	2.05 (1.40–2.99)	< 0.001^***^	2.03 (1.38–2.98)	< 0.001^***^	1.65 (1.10–2.47)	0.016^*^
Walking speed trajectory								
Stable group	1.00 (Reference)	–	1.00 (Reference)	–	1.00 (Reference)	–	1.00 (Reference)	–
Low–stable group	2.90 (1.95–4.31)	< 0.001^***^	2.49 (1.64–3.78)	< 0.001^***^	2.51 (1.65–3.81)	< 0.001^***^	2.37 (1.55–3.62)	< 0.001^***^
Moderate–steep increasing group	4.86 (3.25–7.24)	< 0.001^***^	3.62 (2.38–5.51)	< 0.001^***^	3.59 (2.36–5.47)	< 0.001^***^	3.17 (2.04–4.94)	< 0.001^***^
High–increasing group	6.09 (3.27–11.34)	< 0.001^***^	4.97 (2.61–9.47)	< 0.001^***^	5.38 (2.81–10.31)	< 0.001^***^	4.54 (2.30–8.94)	< 0.001^***^
*P* for trend	8.90 (5.71–13.87)	< 0.001^***^	6.36 (3.91–10.35)	< 0.001^***^	6.55 (4.00–10.72)	< 0.001^***^	5.50 (3.24–9.35)	< 0.001^***^
SHARE								
Log Walking speed	1.15 (0.93–1.42)	0.211	1.11 (0.89–1.38)	0.346	1.11 (0.89–1.37)	0.346	1.06 (0.87–1.30)	0.569
Walking speed trajectory								
Stable group	1.00 (Reference)	–	1.00 (Reference)	–	1.00 (Reference)	–	1.00 (Reference)	–
Low–stable group	1.06 (0.74–1.54)	0.737	1.07 (0.74–1.56)	0.717	1.07 (0.73–1.56)	0.735	1.01 (0.69–1.47)	0.972
Moderate–steep increasing group	2.21 (1.46–3.34)	< 0.001^***^	2.15 (1.40–3.30)	< 0.001^***^	2.11 (1.37–3.24)	< 0.001^***^	1.88 (1.20–2.92)	0.005^**^
High–increasing group	1.44 (0.71–2.93)	0.308	1.45 (0.71–2.99)	0.309	1.47 (0.71–3.03)	0.295	1.33 (0.64–2.76)	0.445
*P* for trend	2.07 (1.34–3.21)	0.001^**^	2.05 (1.30–3.23)	0.002^**^	2.03 (1.29–3.21)	0.002^**^	1.81 (1.13–2.90)	0.014^*^

Model 1: Crude.

Model 2: Adjust: Age, Sex, Race, Education, marital status, Wealth, BMI.

Model 3: Adjust: Age, Sex, Race, Education, marital status, Wealth, BMI, Hypertension, Diabetes, Drinking status, Smoking status.

Model 4: Adjust: Age, Sex, Race, Education, marital status, Wealth, BMI, Hypertension, Diabetes, Drinking status, Smoking status, Physical activity, Cardiovascular disease, Cancer.

**Figure 3:**
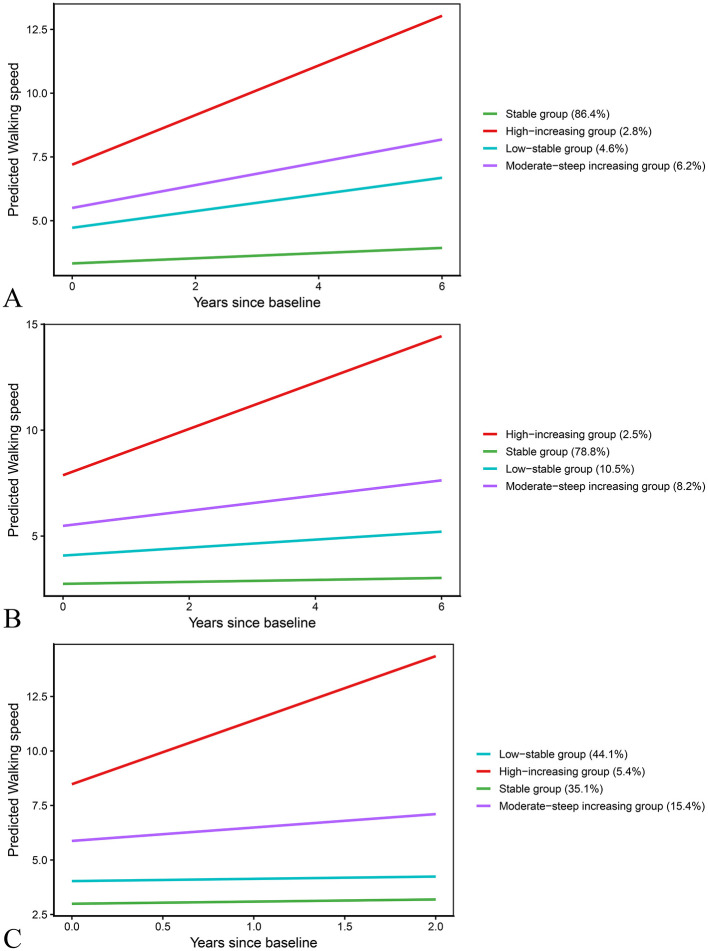
Trajectories of walking speed identified using group-based trajectory modeling (GBTM) in the study population: **(A)** HRS cohort; **(B)** ELSA cohort; **(C)** SHARE cohort.

Significance: ^*^P < 0.05, ^**^P < 0.01, ^***^P < 0.001.

(Supplementary Tables 1–3) present the detailed trajectory model selection process for each cohort, including comparisons of competing trajectory solutions with different group numbers and polynomial specifications, as well as corresponding AIC values, BIC values, log-likelihood statistics, mean posterior probabilities, and group membership proportions. GBTM identified four longitudinal walking speed patterns: stable, low-stable, moderate-steep increasing, and high-increasing (Figure 3), (Supplementary Table 1). Using the stable group as a reference, a progressive escalation in fracture risk emerged from the low-stable to the high-increasing groups. After adjusting for confounders in the HRS cohort, the HRs were 1.82 (95% CI: 1.12–2.96, *P* = 0.015) for the low-stable group, 2.00 (95% CI: 1.32–3.05, *P* = 0.001) for the moderate-steep increasing group, and 2.56 (95% CI: 1.49–4.39, *P* < 0.001) for the high-increasing group. Corresponding adjusted HRs in the ELSA cohort were 2.37 (95% CI: 1.55–3.62, *P* < 0.001), 3.17 (95% CI: 2.04–4.94, *P* < 0.001), and 4.54 (95% CI: 2.30–8.94, *P* < 0.001) (Table 4).

Trend analyses indicated that each successive magnitude increase in the walking speed trajectory significantly amplified fracture risk. This corresponded to a 3.29-fold increase per trajectory level in the HRS cohort (adjusted HR = 4.29, 95% CI: 2.26–8.12, *P* < 0.001) and a 4.50-fold increase in the ELSA cohort (adjusted HR = 5.50, 95% CI: 3.24–9.35, *P* < 0.001). Conversely, in the SHARE cohort, while log-transformed walking speed exhibited a positive correlation post-adjustment, it did not reach statistical significance (adjusted HR = 1.06, 95% CI: 0.87–1.30, *P* = 0.569). Although all three elevated-trajectory groups showed higher risks relative to the stable group in the SHARE cohort, only the moderate-steep increasing group achieved statistical significance. Nonetheless, the trend analysis corroborated that each incremental trajectory level significantly augmented the fracture risk (adjusted HR = 1.81, 95% CI: 1.13–2.90, *P* = 0.014) (Table 4).

**Figure 4:**
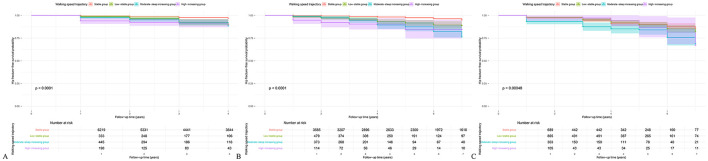
Kaplan–Meier curves illustrating differences in survival probabilities across walking speed trajectory groups: **(A)** HRS cohort; **(B)** ELSA cohort; **(C)** SHARE cohort.

Differences in fracture risk across trajectory groups were further evaluated using Kaplan-Meier survival analysis. In the HRS cohort (mean follow-up of 8 years), significant disparities in event-free survival probabilities were detected among the trajectories (log-rank *P* < 0.0001). The high-increasing group demonstrated the lowest survival probability, declining to approximately 0.88 by year 8. This was followed by the moderate-steep increasing and low-stable groups, with survival probabilities of approximately 0.92 and 0.93, respectively. The stable group maintained optimal skeletal safety, with its curve remaining near 1.00. Notably, the high-increasing group diverged from the others as early as year 2, with disparities widening progressively over time. This suggests that sharp escalations in walking speed may signal underlying deteriorations in skeletal health or an augmented fall risk (Figure 4A).

External validation in the ELSA cohort (14-year follow-up) closely aligned with the HRS findings (log-rank *P* < 0.0001). The high-increasing group exhibited the lowest event-free survival probability (~0.78 at 14 years), while the moderate-steep increasing and low-stable groups displayed intermediate risks (~0.85 and ~0.89, respectively). The stable group sustained a 14-year survival probability exceeding 0.95. The risk gradient patterns were highly consistent between the cohorts (Figure 4B). Similar validation in the SHARE cohort (14-year follow-up) confirmed these statistical disparities (log-rank *P* < 0.0001). The lowest event-free survival probability was again seen in the high-increasing group (~0.65 at 14 years), whereas the stable group maintained a survival probability above 0.80. Across all three cohorts, the maximum risk differential was consistently observed between the high-increasing and stable groups (Figure 4C).

### 3.3 Competing risk relationship accounting for mortality

Fine-Gray competing risk regression models, which treated mortality as a competing event, yielded results consistent with the Cox models. Post-adjustment, log-transformed walking speed was independently associated with an elevated sHR for new-onset hip fractures (HRS: adjusted sHR = 1.61, 95% CI: 1.13–2.29, *P* = 0.009; ELSA: adjusted sHR = 1.64, 95% CI: 1.10–2.46, *P* = 0.016; SHARE: adjusted sHR = 1.06, 95% CI: 0.85–1.32, *P* = 0.610).

Following GBTM categorization and covariate adjustment, consistent dose-response trends emerged in the HRS and ELSA cohorts. Relative to the stable group, risks in the HRS cohort increased by 81% in the low-stable group (sHR = 1.81, 95% CI: 1.13–2.89, *P* = 0.014), 99% in the moderate-steep increasing group (sHR = 1.99, 95% CI: 1.31–3.03, *P* = 0.001), and 154% in the high-increasing group (sHR = 2.54, 95% CI: 1.45–4.43, *P* = 0.001). In the ELSA cohort, corresponding risk increments were 134% (sHR = 2.34, 95% CI: 1.53–3.58, *P* < 0.001), 211% (sHR = 3.11, 95% CI: 1.96–4.91, *P* < 0.001), and 344% (sHR = 4.44, 95% CI: 2.14–9.21, *P* < 0.001). In the SHARE cohort, only the moderate-steep increasing group exhibited a significantly elevated risk compared to the stable group (sHR = 1.85, 95% CI: 1.20–2.86, *P* = 0.006).

Trend analyses based onthe Fine-Gray regression confirmed that each incremental trajectory level significantly amplified fracture risk. Risk increased 3.23-fold in the HRS cohort (adjusted sHR = 4.23, 95% CI: 2.19–8.16, *P* < 0.001), 4.35-fold in the ELSA cohort (adjusted sHR = 5.35, 95% CI: 3.05–9.40, *P* < 0.001), and 78% in the SHARE cohort (adjusted sHR = 1.78, 95% CI: 1.11–2.86, *P* = 0.016). The magnitude and direction of the competing risk analyses mirrored the Cox models, indicating that mortality as a competing risk did not substantially compromise these associations (Table 5).

**Table 5:** Fine-Gray regression analyses assessing whether the associations between walking speed, its trajectory patterns, and the risk of incident hip fracture are influenced by mortality.

Exposure Variable	Model 1		Model 2		Model 3		Model 4	
	sHR (95% CI)	*P*-value	sHR (95% CI)	*P*-value	sHR (95% CI)	*P*-value	sHR (95% CI)	*P*-value
HRS								
Log walking speed	2.83 (2.12–3.78)	< 0.001^***^	2.03 (1.48–2.78)	< 0.001^***^	1.87 (1.35–2.59)	< 0.001^***^	1.61 (1.13–2.29)	0.009^**^
Walking speed trajectory								
Stable group	1.00 (Reference)	–	1.00 (Reference)	–	1.00 (Reference)	–	1.00 (Reference)	–
Low–stable group	2.80 (1.77–4.43)	< 0.001^***^	2.20 (1.37–3.55)	0.001^**^	2.05 (1.27–3.31)	0.003^**^	1.81 (1.13–2.89)	0.014^*^
Moderate–steep increasing group	3.41 (2.31–5.04)	< 0.001^***^	2.52 (1.68–3.78)	< 0.001^***^	2.26 (1.50–3.42)	< 0.001^***^	1.99 (1.31–3.03)	0.001^**^
High–increasing group	4.42 (2.65–7.36)	< 0.001^***^	3.31 (1.94–5.68)	< 0.001^***^	3.07 (1.78–5.27)	< 0.001^***^	2.54 (1.45–4.43)	0.001^**^
*P* for trend	11.73 (6.65–20.68)	< 0.001^***^	6.72 (3.58–12.62)	< 0.001^***^	5.61 (2.95–10.66)	< 0.001^***^	4.23 (2.19–8.16)	< 0.001^***^
ELSA								
Log walking speed	2.65 (1.96–3.57)	< 0.001^***^	2.04 (1.43–2.91)	< 0.001^***^	2.02 (1.41–2.90)	< 0.001^***^	1.64 (1.10–2.46)	0.016^*^
Walking speed trajectory								
Stable group	1.00 (Reference)	–	1.00 (Reference)	–	1.00 (Reference)	–	1.00 (Reference)	–
Low–stable group	2.89 (1.95–4.27)	< 0.001^***^	2.47 (1.63–3.75)	< 0.001^***^	2.48 (1.63–3.77)	< 0.001^***^	2.34 (1.53–3.58)	< 0.001^***^
Moderate–steep increasing group	4.79 (3.24–7.09)	< 0.001^***^	3.56 (2.37–5.33)	< 0.001^***^	3.52 (2.33–5.30)	< 0.001^***^	3.11 (1.96–4.91)	< 0.001^***^
High–increasing group	6.03 (3.22–11.29)	< 0.001^***^	4.89 (2.53–9.45)	< 0.001^***^	5.27 (2.73–10.18)	< 0.001^***^	4.44 (2.14–9.21)	< 0.001^***^
*P* for trend	8.77 (5.73–13.42)	< 0.001^***^	6.21 (3.87–9.98)	< 0.001^***^	6.37 (3.94–10.31)	< 0.001^***^	5.35 (3.05–9.40)	< 0.001^***^
SHARE								
Log Walking speed	1.14 (0.89–1.47)	0.300	1.11 (0.86–1.42)	0.420	1.11 (0.86–1.42)	0.430	1.06 (0.85–1.32)	0.610
Walking speed trajectory								
Stable group	1.00 (Reference)	–	1.00 (Reference)	–	1.00 (Reference)	–	1.00 (Reference)	–
Low–stable group	1.06 (0.74–1.53)	0.740	1.07 (0.73–1.56)	0.720	1.07 (0.73–1.55)	0.740	1.01 (0.69–1.47)	0.970
Moderate–steep increasing group	2.17 (1.45–3.26)	< 0.001^***^	2.12 (1.39–3.23)	< 0.001^***^	2.08 (1.36–3.16)	< 0.001^***^	1.85 (1.20–2.86)	0.006^**^
High–increasing group	1.44 (0.74–2.80)	0.290	1.45 (0.73–2.87)	0.290	1.46 (0.73–2.91)	0.280	1.33 (0.65–2.72)	0.440
*P* for trend	2.04 (1.33–3.15)	0.001^**^	2.02 (1.29–3.16)	0.002^**^	2.00 (1.28–3.13)	0.002^**^	1.78 (1.11–2.86)	0.016^*^

Model 1: Crude

Model 2: Adjust: Age, Sex, Race, Education, marital status, Wealth, BMI.

Model 3: Adjust: Age, Sex, Race, Education, marital status, Wealth, BMI, Hypertension, Diabetes, Drinking status, Smoking status

Model 4: Adjust: Age, Sex, Race, Education, marital status, Wealth, BMI, Hypertension, Diabetes, Drinking status, Smoking status, Physical activity, Cardiovascular disease, Cancer

Significance: ^*^P < 0.05, ^**^P < 0.01, ^***^P < 0.001.

### 3.4 Discrete-time logistic regression analysis

A discrete-time logistic regression model was fitted using the follow-up wave as the temporal unit, with data restructured into a person-period format. Both walking speed and its trajectories were significantly associated with fracture incidence during each follow-up interval. Due to data structure limitations precluding a person-period conversion in the SHARE cohort, this analysis was restricted to the HRS and ELSA databases. In fully adjusted models, log-transformed walking speed exhibited a significant positive correlation with fracture risk (HRS: adjusted OR = 1.63, 95% CI: 1.13–2.31, *P* = 0.008; ELSA: adjusted OR = 1.76, 95% CI: 1.12–2.69, *P* = 0.011). Compared to the stable group, participants in all escalating trajectory groups demonstrated an elevated fracture risk in both datasets. These findings aligned consistently in direction and magnitude with prior models, affirming the robustness of the results across diverse temporal modeling strategies (Table 6).

**Table 6:** Discrete-time logistic regression analyses examining the associations between walking speed, its trajectory patterns, and the risk of incident hip fracture among individuals with osteoporosis.

Exposure variable	Model 1		Model 2		Model 3		Model 4	
	OR (95% CI)	*P*-value	OR (95% CI)	*P*-value	OR (95% CI)	*P*-value	OR (95% CI)	*P*-value
HRS								
Log walking speed	2.89 (2.09–3.93)	< 0.001^***^	2.03 (1.43–2.84)	< 0.001^***^	1.89 (1.33–2.65)	< 0.001^***^	1.63 (1.13–2.31)	0.008^**^
Walking speed trajectory								
Stable group	1.00 (Reference)	–	1.00 (Reference)	–	1.00 (Reference)	–	1.00 (Reference)	–
Low–stable group	2.84 (1.70–4.48)	< 0.001^***^	2.19 (1.29–3.52)	0.002^**^	2.06 (1.21–3.33)	0.005^**^	1.83 (1.07–2.97)	0.019^*^
Moderate–steep increasing group	3.38 (2.18–5.02)	< 0.001^***^	2.44 (1.55–3.71)	< 0.001^***^	2.22 (1.41–3.37)	< 0.001^***^	1.96 (1.23–2.99)	0.003^**^
High–increasing group	4.67 (2.66–7.65)	< 0.001^***^	3.49 (1.95–5.84)	< 0.001^***^	3.23 (1.81–5.42)	< 0.001^***^	2.69 (1.49–4.55)	< 0.001^***^
*P* for trend	12.11 (6.59–21.69)	< 0.001^***^	6.70 (3.47–12.64)	< 0.001^***^	5.70 (2.95–10.80)	< 0.001^***^	4.33 (2.21–8.34)	< 0.001^***^
ELSA								
Log Walking speed	2.65 (1.83–3.77)	< 0.001^***^	2.14 (1.41–3.17)	< 0.001^***^	2.13 (1.40–3.17)	< 0.001^***^	1.76 (1.12–2.69)	0.011^*^
Walking speed trajectory								
Stable group	1.00 (Reference)	–	1.00 (Reference)	–	1.00 (Reference)	–	1.00 (Reference)	–
Low–stable group	2.82 (1.81–4.26)	< 0.001^***^	2.52 (1.58–3.90)	< 0.001^***^	2.52 (1.58–3.92)	< 0.001^***^	2.41 (1.50–3.76)	< 0.001^***^
Moderate–steep increasing group	4.62 (2.92–7.07)	< 0.001^***^	3.59 (2.22–5.62)	< 0.001^***^	3.58 (2.21–5.61)	< 0.001^***^	3.22 (1.94–5.19)	< 0.001^***^
High–increasing group	6.70 (3.35–12.13)	< 0.001^***^	5.87 (2.87–10.97)	< 0.001^***^	6.15 (2.99–11.56)	< 0.001^***^	5.33 (2.51–10.43)	< 0.001^***^
P for trend	8.88 (5.44–14.20)	< 0.001^***^	6.80 (3.99–11.44)	< 0.001^***^	6.93 (4.04–11.70)	< 0.001^***^	5.99 (3.35–10.61)	< 0.001^***^

Model 1: crude.

Model 2: Adjust: Age, Sex, Race, Education, marital status, Wealth, BMI.

Model 3: Adjust: Age, Sex, Race, Education, marital status, Wealth, BMI, Hypertension, Diabetes, Drinking status, Smoking status.

Model 4: Adjust: Age, Sex, Race, Education, marital status, Wealth, BMI, Hypertension, Diabetes, Drinking status, Smoking status, Physical activity, Cardiovascular disease, Cancer.

Significance: ^*^P < 0.05, ^**^P < 0.01, ^***^P < 0.001.

### 3.5 Subgroup analysis

**Figure 5:**
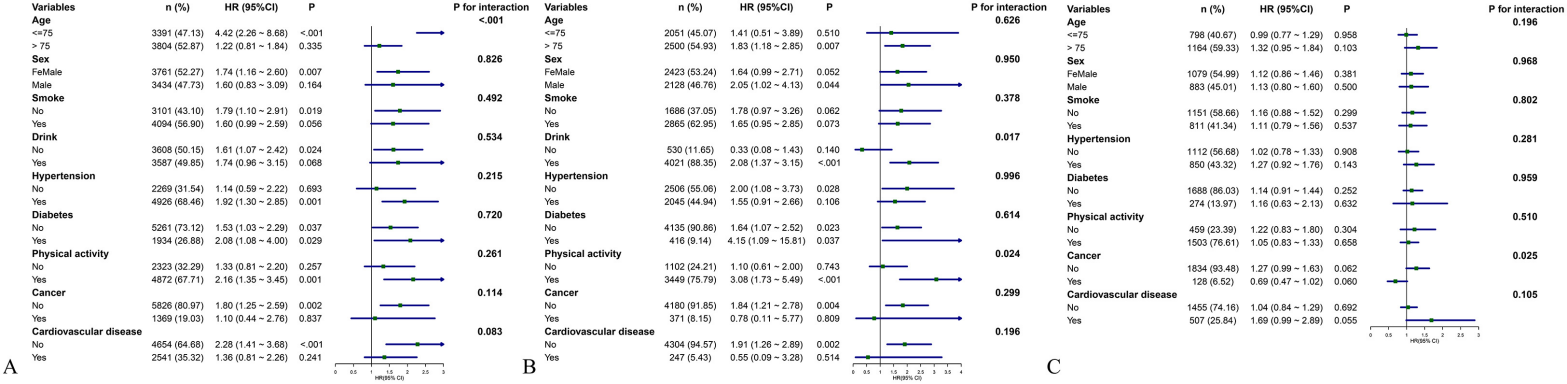
Forest plots showing the associations between walking speed and the risk of new-onset Hip fracture across different subgroups in each cohort: **(A)** HRS cohort; **(B)** ELSA cohort; **(C)** SHARE cohort.

Subgroup analyses evaluated whether the established associations varied across distinct demographic and clinical populations. Within the HRS cohort, the positive correlation remained consistent across most strata. Stratified analyses revealed a more pronounced association in individuals aged ≤ 75 years (HR = 4.42, 95% CI: 2.26–8.68, *P* < 0.001), with a statistically significant interaction term (*P* for interaction < 0.001). The association was also robust among females (HR = 1.74, *P* = 0.007), non-smokers (HR = 1.79, *P* = 0.019), non-drinkers (HR = 1.61, *P* = 0.024), and individuals with hypertension (HR = 1.92, *P* = 0.001), regular physical activity (HR = 2.16, *P* = 0.001), and an absence of cancer (HR = 1.80, *P* = 0.002) or CVD (HR = 2.28, *P* < 0.001). Interaction tests across most other subgroups were non-significant (all *P* for interaction > 0.05), suggesting overall stability (Figure 5A).

In the ELSA cohort, findings were broadly similar. The association was significant in individuals aged > 75 years (HR = 1.83, 95% CI: 1.18–2.85, *P* = 0.007), though non-significant in those ≤ 75 years. The correlation remained stable among males (HR = 2.05, *P* = 0.044), drinkers (HR = 2.08, *P* < 0.001), and individuals without hypertension (HR = 2.00, *P* = 0.028), physically active participants (HR = 3.08, *P* < 0.001), and those lacking cancer (HR = 1.84, *P* = 0.004) or CVD (HR = 1.91, *P* = 0.002). Notably, significant interactions were detected for drinking status (*P* for interaction = 0.017) and physical activity (*P* for interaction = 0.024), indicating potential moderating effects (Figure 5B). In the SHARE cohort, the relationship remained stable across subpopulations, although trend analyses suggested cancer might exert a modifying effect on fracture risk (*P* for interaction = 0.025) (Figure 5C).

### 3.6 Linear and non-linear relationships

**Figure 6:**
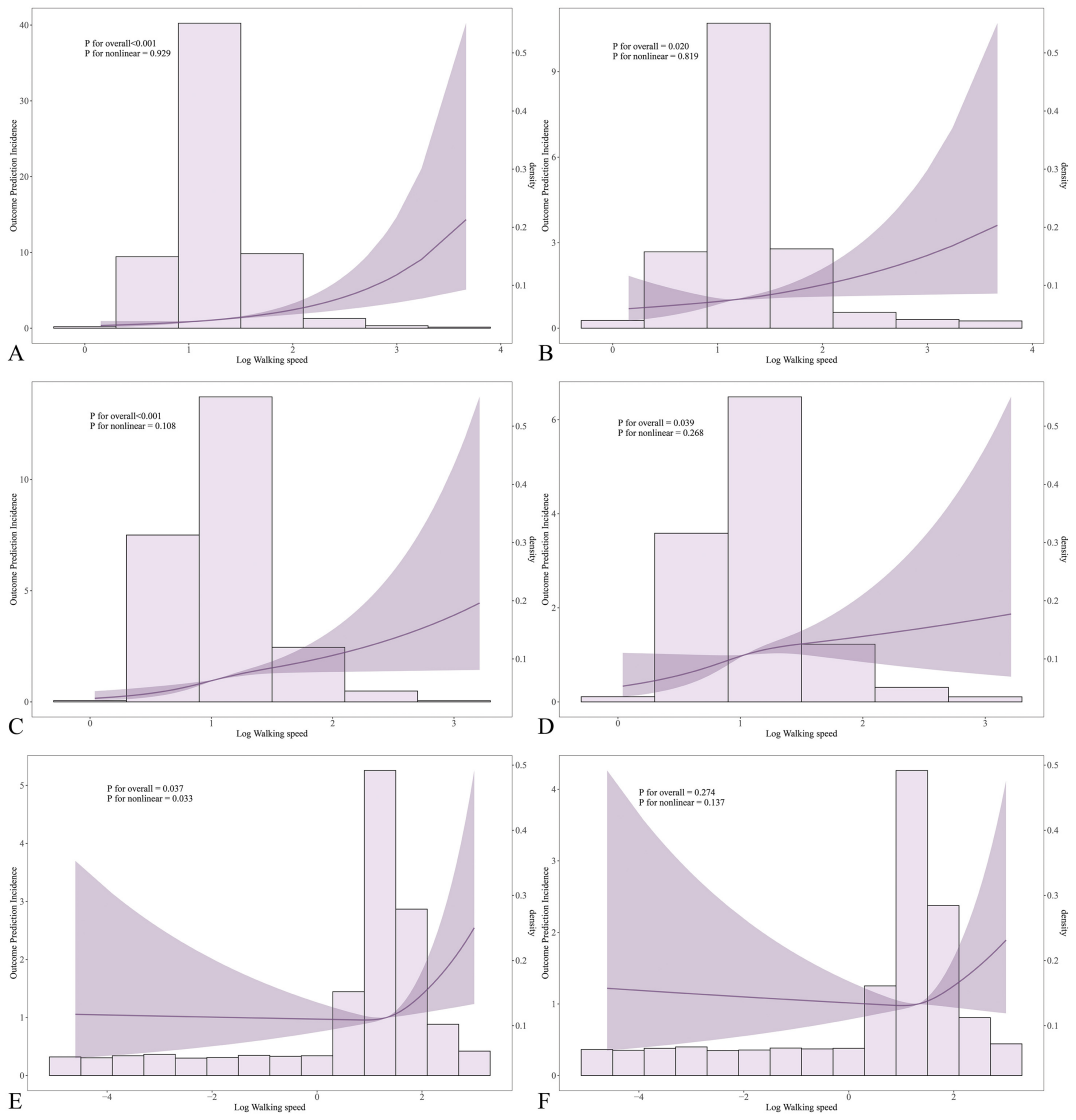
Restricted cubic spline analyses illustrating the linear and nonlinear relationships between walking speed and the risk of new-onset Hip fracture: **(A)** unadjusted model in the HRS cohort; **(B)** fully adjusted model in the HRS cohort; **(C)** unadjusted model in the ELSA cohort; **(D)** fully adjusted model in the ELSA cohort; **(E)** unadjusted model in the SHARE cohort; **(F)** fully adjusted model in the SHARE cohort.

RCS analysesvisually characterized the dose-response relationship between walking speed and fracture risk. Prior to covariate adjustment, the relationship was linear in both the HRS and ELSA cohorts (HRS: *P* for non-linear = 0.929, *P* for overall < 0.001; ELSA: *P* for non-linear = 0.108, *P* for overall < 0.001) (Figures 6A, C), whereas a non-linear relationship was observed in the SHARE cohort (*P* for non-linear = 0.033, *P* for overall < 0.001) (Figure 6E). Following adjustments, the linear relationship remained robust across all three cohorts (HRS: *P* for non-linear = 0.819; ELSA: *P* for non-linear = 0.268; SHARE: *P* for non-linear = 0.137) (Figures 6B, D,F).

### 3.7 Mediation analysis

**Figure 7:**
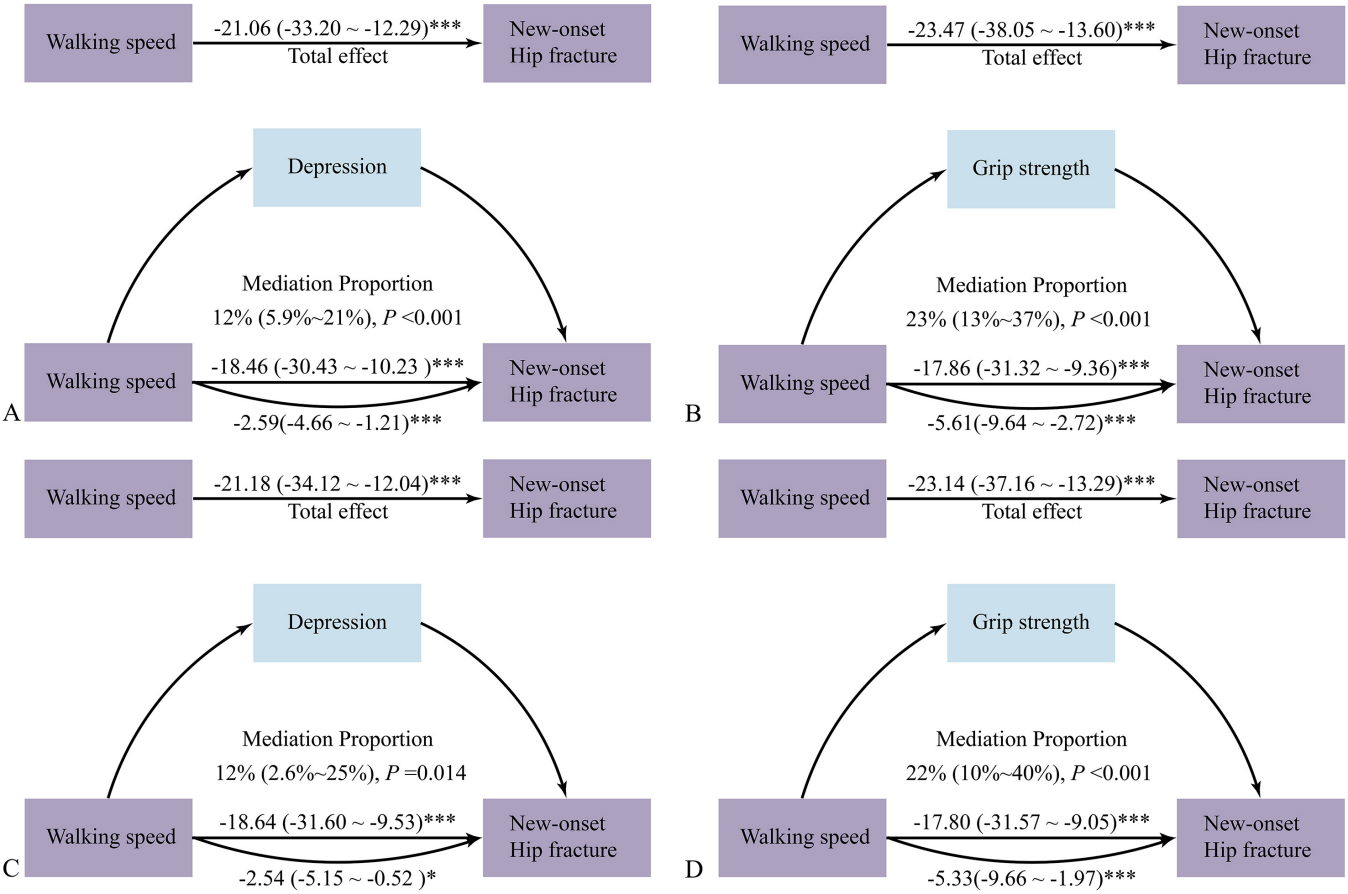
Mediation analyses evaluating the mediating roles of muscle strength and depression in the association between walking speed and the risk of incident hip fracture: **(A, B)** HRS cohort; **(C, D)** ELSA cohort. ^*^ and ^***^ denote statistical significance at the levels of *p* < 0.05 and *p* < 0.001.

Figure 6 illustrates the mediating roles of depression and muscle strength, both of which acted as partial mediators between walking speed and fracture risk. In the HRS database, depression mediated approximately 12% of the total effect (Figure 7A), and muscle strength mediated roughly 23% (Figure 7B). Correspondingly, in the ELSA database, depression and muscle strength mediated approximately 12% (Figure 7C) and 22% (Figure 7D) of the total effect, respectively. Both direct and total effects were statistically significant across these cohorts. Because the baseline data for the SHARE cohort lacked variables for muscle strength and depression, it was excluded from the mediation analysis module. These mediation findings should be interpreted cautiously because the observational study design cannot fully establish causal temporality or exclude residual confounding.

### 3.8 Sensitivity analysis

In sensitivity analyses further adjusting for fall history, the associations between walking speed trajectories and incident hip fracture remained generally consistent with the primary Cox regression analyses, indicating that the observed associations were relatively robust to additional adjustment for fall-related risk (Supplementary Table 5).

## 4 Discussion

Utilizingthree large-scale longitudinal cohorts (HRS, ELSA, and SHARE), this study systematically evaluated the association between walking speed, its temporal trajectories, and the risk of incident hip fracture among individuals with osteoporosis. The primary finding was that both higher baseline walking speed and persistently increasing walking speed trajectories were associated with a significantly elevated risk of hip fracture. Importantly, this association demonstrated a clear dose–response relationship and remained robust across multiple statistical approaches, including Cox regression, competing-risk analyses, and discrete-time models. Replication across independent cohorts further supports the robustness and external validity of the findings.

Notably, these findings differ from much of the existing literature showing that slower gait speed predicts frailty, falls, disability, and mortality in older adults. However, most previous studies were conducted in general aging populations and primarily focused on functional decline rather than fracture risk among individuals with osteoporosis. In contrast, the present study specifically examined osteoporotic individuals, in whom fracture occurrence may be influenced not only by physical performance but also by environmental exposure, mobility behavior, and underlying skeletal fragility. Therefore, faster walking speed in this population should not necessarily be interpreted as uniformly protective.

Higher walking speed is generally considered a marker of better physical performance and neuromuscular function. Therefore, the observed positive association between higher walking speed and increased hip fracture risk may initially appear counterintuitive. One possible explanation is the “fall exposure” hypothesis. Individuals with higher walking speed are more likely to remain socially and physically active, increasing exposure to environmental hazards such as uneven surfaces, stairs, or outdoor obstacles. In the context of osteoporosis, even relatively minor falls may lead to hip fractures because of impaired bone strength and compromised skeletal microarchitecture (16, 17). In addition, the observed association may partly reflect behavioral or survival-related mechanisms. Older adults with preserved mobility may survive longer and maintain higher levels of daily activity, thereby accumulating greater long-term exposure to fall risk. Conversely, individuals with severe frailty or markedly reduced mobility may restrict activity and environmental exposure, potentially lowering short-term fracture occurrence despite poorer overall health status (18, 19). Importantly, although the analyses adjusted for physical activity and multiple health-related covariates, residual confounding by unmeasured mobility behaviors, environmental exposure, or functional reserve cannot be completely excluded. Therefore, the present findings should be interpreted cautiously and should not be considered evidence that faster walking speed directly causes hip fracture.

Another important findingof this study is that longitudinal walking speed trajectories captured dynamic changes in mobility over time and identified distinct trajectory patterns associated with incident hip fracture risk. Traditional cross-sectional assessments capture only a single time point and may not adequately reflect longitudinal functional changes. In contrast, trajectory modeling characterized long-term mobility patterns and demonstrated a monotonic increase in hip fracture risk across progressively faster trajectory groups. Notably, individuals with persistently increasing walking speed exhibited the highest fracture risk. These findings suggest that higher or persistently increasing walking speed in individuals with osteoporosis may partly reflect greater activity-related environmental exposure and mobility-related fall opportunity rather than a direct harmful effect of faster walking speed itself. In addition, survival-related selection mechanisms may also contribute to the observed associations, as individuals with relatively preserved mobility may remain active for longer periods and consequently accumulate greater exposure to fall risk over time. Therefore, longitudinal assessment of walking speed trajectories may provide additional insight into mobility-related fracture risk patterns beyond conventional static functional measurements (20, 21).

Subgroup analyses revealed clinically meaningful heterogeneity. In the HRS cohort, the association was stronger among individuals aged ≤ 75 years, which may reflect higher activity levels and greater environmental exposure in younger older adults. In contrast, in the ELSA cohort, the association was more pronounced among individuals aged >75 years, potentially due to accelerated bone loss, impaired balance, and increased frailty in advanced age (22, 23). Sex-stratified analyses indicated a stronger association among women in the HRS cohort, possibly attributable to postmenopausal estrogen deficiency and consequent reductions in bone mineral density, which increase susceptibility to fracture at a given level of fall risk (24, 25). Significant interactions with alcohol consumption and physical activity, particularly in the ELSA cohort, further suggest that modifiable behavioral factors may influence underlying risk pathways, underscoring the multifactorial nature of osteoporotic fractures (26, 27).

Mediation analysis provides additional exploratory insight into potential pathways linking walking speed and hip fracture risk. Depression and muscle strength appeared to partially explain the observed associations. Depression accounted for approximately 12% of the total effect, potentially reflecting impaired attention, delayed reaction time, reduced motivation, and psychomotor slowing, all of which may influence mobility and fall susceptibility (28, 29). Muscle strength accounted for a larger proportion of the association, suggesting that neuromuscular function may contribute to the relationship between walking performance and fracture vulnerability (30, 31). However, these findings should be interpreted cautiously because mediation analyses in observational longitudinal studies cannot fully establish causal relationships or temporal ordering. Residual confounding, bidirectional associations, and unmeasured behavioral or health-related factors may still influence the observed mediation effects.

Several limitations should be considered. First, osteoporosis and hip fracture were identified based on self-reported physician diagnoses, which may have introduced misclassification bias and potentially underestimated the true prevalence and incidence of these conditions. However, standardized imaging confirmation and medical record validation were not consistently available across HRS, ELSA, and SHARE, and self-reported physician diagnoses were therefore used to ensure harmonized definitions across cohorts. Previous epidemiological studies using these databases have similarly relied on self-reported fracture-related outcomes, and prior validation studies have demonstrated acceptable reliability of self-reported major fracture events, particularly hip fractures, among older adults (3). Moreover, the consistency of the observed associations across three independent international cohorts partially reduces the likelihood that the findings were solely driven by cohort-specific outcome misclassification. In addition, the consistency of findings across three independent international cohorts may partially reduce concerns regarding cohort-specific reporting bias or outcome misclassification. Second, residual confounding cannot be completely excluded because several clinically important fracture-related variables were not consistently available across the three cohorts. Although additional sensitivity analyses further adjusting for fall history yielded results generally consistent with the primary analyses, some important variables, including anti-osteoporosis medication use, corticosteroid exposure, bone mineral density, frailty status, and detailed medication data, were unavailable or not harmonized across datasets. Consequently, some of the observed associations may still partly reflect unmeasured clinical, behavioral, or treatment-related factors. Therefore, the findings should be interpreted cautiously within the context of observational epidemiological research. Third, both cohorts used relatively short walking distance protocols, which differ from the standardized 4-meter assessment commonly recommended, potentially limiting measurement precision and comparability with other studies (7). Fourth, although walking speed trajectories were assessed before outcome follow-up and participants with baseline hip fracture were excluded to strengthen temporal ordering, the observational design cannot completely eliminate the possibility of reverse causation. Subclinical health deterioration, progressive frailty, or other underlying conditions may have influenced walking speed patterns before the occurrence of clinically recognized hip fracture. Therefore, the observed associations should be interpreted as longitudinal epidemiological associations rather than definitive evidence of a causal relationship. Fifth, the mediation analyses were exploratory in nature, and the observational design limited our ability to establish definitive causal mediation pathways between walking speed, depression, muscle strength, and hip fracture risk. Additionally, a considerable number of participants, particularly in the SHARE cohort, were excluded because of missing walking speed measurements required for trajectory construction. Comparison analyses demonstrated that included and excluded participants differed significantly in several demographic and clinical characteristics, including age, body mass index, cardiovascular disease, hypertension, and smoking status, indicating the possibility of selection bias and potentially limiting the generalizability of the findings. Because trajectory modeling required repeated walking speed assessments across the first three waves, participants without sufficient longitudinal gait data could not be reliably classified into trajectory groups. Finally, the lack of uniformly available dual-energy X-ray absorptiometry data limits further exploration of interactions with bone mineral density and bone quality (32). Furthermore, detailed measures of mobility behavior, environmental exposure, and objective fall frequency were not uniformly available across cohorts, limiting further exploration of the biological and behavioral mechanisms underlying the observed associations.

Overall, the present study highlights the potential importance of longitudinal mobility assessment in fracture risk evaluation among older adults with osteoporosis. However, the findings should be interpreted cautiously given the observational nature of the study and the potential influence of residual confounding and behavioral selection mechanisms. Rather than suggesting that faster walking speed is harmful, the results may indicate that greater mobility and environmental exposure interact with skeletal fragility to influence fracture risk. Accordingly, the present findings should be interpreted primarily as longitudinal associations rather than definitive causal relationships. Future studies incorporating detailed fracture-related clinical variables, objective fall assessments, medication exposure data, and bone mineral density measurements are warranted to further validate and clarify the observed associations.

## 5 Conclusions

In summary, walking speed and its longitudinal trajectories are important predictors of incident hip fracture among individuals with osteoporosis. A stepwise increase in fracture risk was observed with higher levels and greater increases in walking speed over time. These findings highlight the importance of incorporating dynamic indicators of physical function into conventional fracture risk assessment. Future studies should further elucidate the underlying mechanisms and determine whether interventions targeting walking function can reduce fracture risk, thereby informing clinical risk stratification and prevention strategies.

## Data Availability

The datasets presented in this study can be found in online repositories. The names of the repository/repositories and accession number(s) can be found in the article/Supplementary material.
