# Supplementary material for: Longitudinal trajectories of walking speed and risk of incident hip fracture in osteoporosis: a group-based trajectory modeling analysis from HRS, ELSA and SHARE
Source: Front Public Health. 2026 Jul 2;14:1857692. doi: 10.3389/fpubh.2026.1857692 (PMC13372962; doi:10.3389/fpubh.2026.1857692)
Supplement: Supplementary file 1 [file Supplementary_file_1.docx]

Supplementary Table 1 Model fit statistics for competing trajectory models in HRS, ELSA, and SHARE cohorts

| **Model** | **AIC** | **BIC** | **Log-Likelihood** | **Smallest Group (%)** | **Mean Posterior Probability** |
| --- | --- | --- | --- | --- | --- |
| HRS |  |  |  |  |  |
| 1-class | 129,855.4 | 129,896.7 | -64,921.72 | 100.0 |  |
| 2-class | 123,386.2 | 123,455.0 | -61,683.11 | 32.4 | 0.82 |
| 3-class | 122,768.4 | 122,892.3 | -61,377.80 | 11.7 | 0.86 |
| 4-class | 122,783.6 | 122,879.9 | -61,366.23 | 7.3 | 0.89 |
| Elsa |  |  |  |  |  |
| 1-class | 77,524.67 | 77,563.21 | -38,756.34 | 100.0 |  |
| 2-class | 70,709.94 | 70,774.17 | -35,344.97 | 28.5 | 0.81 |
| 3-class | 69,869.73 | 69,959.65 | -34,920.86 | 10.8 | 0.85 |
| 4-class | 69,699.57 | 69,815.19 | -34,831.79 | 6.5 | 0.88 |
| SHARE |  |  |  |  |  |
| 1-class | 20,268.60 | 20,302.09 | -10,128.30 | 100.0 |  |
| 2-class | 17,570.60 | 17,626.41 | -8,775.30 | 24.1 | 0.80 |
| 3-class | 17,199.03 | 17,277.18 | -8,585.52 | 9.9 | 0.84 |
| 4-class | 17,142.09 | 17,242.56 | -8,553.04 | 5.8 | 0.87 |
| AIC: Akaike Information Criterion; BIC: Bayesian Information Criterion.  The optimal trajectory model was selected based on lower BIC values, adequate posterior classification probability (≥0.70), clinically interpretable trajectories, and minimum subgroup size >5%. | | | | | |
