# Supplementary material for: Longitudinal trajectories of walking speed and risk of incident hip fracture in osteoporosis: a group-based trajectory modeling analysis from HRS, ELSA and SHARE
Source: Front Public Health. 2026 Jul 2;14:1857692. doi: 10.3389/fpubh.2026.1857692 (PMC13372962; doi:10.3389/fpubh.2026.1857692)
Supplement: Supplementary file 2 [file Supplementary_file_2.docx]

Supplementary Table 2 Polynomial specifications and trajectory characteristics of the final four-group GBTM models

| **Cohort** | **Trajectory Group** | **Polynomial Order** | **Estimated Proportion (%)** | **Trajectory Pattern Description** |
| --- | --- | --- | --- | --- |
| HRS | Group 1 | Linear | 41.2 | Stable-low walking speed |
| HRS | Group 2 | Quadratic | 33.5 | Moderate-increasing |
| HRS | Group 3 | Quadratic | 18.0 | High-stable |
| HRS | Group 4 | Cubic | 7.3 | Rapidly increasing |
| Elsa | Group 1 | Linear | 39.4 | Stable-low walking speed |
| Elsa | Group 2 | Quadratic | 35.2 | Moderate-increasing |
| Elsa | Group 3 | Quadratic | 18.9 | High-stable |
| Elsa | Group 4 | Cubic | 6.5 | Rapidly increasing |
| SHARE | Group 1 | Linear | 43.7 | Stable-low walking speed |
| SHARE | Group 2 | Quadratic | 30.6 | Moderate-increasing |
| SHARE | Group 3 | Quadratic | 19.9 | High-stable |
| SHARE | Group 4 | Cubic | 5.8 | Rapidly increasing |
| Polynomial order was determined according to model fit, trajectory stability, and clinical interpretability. | | | | |
