# Supplementary material for: Longitudinal trajectories of walking speed and risk of incident hip fracture in osteoporosis: a group-based trajectory modeling analysis from HRS, ELSA and SHARE
Source: Front Public Health. 2026 Jul 2;14:1857692. doi: 10.3389/fpubh.2026.1857692 (PMC13372962; doi:10.3389/fpubh.2026.1857692)
Supplement: Supplementary file 3 [file Supplementary_file_3.docx]

Supplementary Table 3 Posterior classification quality of the final four-group trajectory models

| **Cohort** | **Trajectory Group** | **Mean Posterior Probability** | **Odds of Correct Classification (OCC)** |
| --- | --- | --- | --- |
| HRS | Group 1 | 0.91 | 12.6 |
| HRS | Group 2 | 0.87 | 10.8 |
| HRS | Group 3 | 0.85 | 9.7 |
| HRS | Group 4 | 0.89 | 11.9 |
| Elsa | Group 1 | 0.90 | 11.8 |
| Elsa | Group 2 | 0.86 | 10.2 |
| Elsa | Group 3 | 0.84 | 9.1 |
| Elsa | Group 4 | 0.88 | 11.1 |
| SHARE | Group 1 | 0.89 | 11.2 |
| SHARE | Group 2 | 0.85 | 9.6 |
| SHARE | Group 3 | 0.83 | 8.8 |
| SHARE | Group 4 | 0.87 | 10.7 |
| Mean posterior probabilities >0.70 indicate acceptable classification accuracy.  Higher OCC values indicate better trajectory assignment performance. | | | |
