# Supplementary material for: Longitudinal trajectories of walking speed and risk of incident hip fracture in osteoporosis: a group-based trajectory modeling analysis from HRS, ELSA and SHARE
Source: Front Public Health. 2026 Jul 2;14:1857692. doi: 10.3389/fpubh.2026.1857692 (PMC13372962; doi:10.3389/fpubh.2026.1857692)
Supplement: Supplementary file 4 [file Supplementary_file_4.docx]

Supplementary Table 4. Comparison of baseline characteristics between included and excluded participants in the SHARE cohort

| **Variable** | **level** | **Excluded** | **Included** | **p** | **test** |
| --- | --- | --- | --- | --- | --- |
| n |  | 29108 | 1308 |  |  |
| Age(year) (mean (SD)) |  | 63.09 (10.19) | 79.92 (3.89) | <0.001 |  |
| BMI (mean (SD)) |  | 26.40 (4.34) | 25.66 (3.66) | <0.001 |  |
| Walking speed (mean (SD)) |  | 4.73 (3.01) | 4.21 (2.51) | <0.001 |  |
| Sex (%) | 0 | 16163 (55.5) | 738 (56.4) | 0.543 |  |
|  | 1 | 12945 (44.5) | 570 (43.6) |  |  |
| Native (%) | 0 | 678 (2.3) | 16 (1.2) | 0.011 |  |
|  | 1 | 28376 (97.7) | 1292 (98.8) |  |  |
| Education (%) | 1 | 14739 (50.6) | 913 (69.8) | <0.001 |  |
|  | 2 | 8814 (30.3) | 243 (18.6) |  |  |
|  | 3 | 5555 (19.1) | 152 (11.6) |  |  |
| Hypertension (%) | 0 | 19925 (68.8) | 764 (58.5) | <0.001 |  |
|  | 1 | 9019 (31.2) | 543 (41.5) |  |  |
| Diabetes (%) | 0 | 26027 (89.9) | 1173 (89.7) | 0.875 |  |
|  | 1 | 2917 (10.1) | 134 (10.3) |  |  |
| Cancer (%) | 0 | 27453 (94.8) | 1225 (93.7) | 0.085 |  |
|  | 1 | 1491 (5.2) | 82 (6.3) |  |  |
| Cardiovascular disease (%) | 0 | 24879 (86.0) | 967 (74.0) | <0.001 |  |
|  | 1 | 4065 (14.0) | 340 (26.0) |  |  |
| smokev (%) | 0 | 15209 (52.5) | 790 (60.4) | <0.001 |  |
|  | 1 | 13740 (47.5) | 518 (39.6) |  |  |
| Physical activity (%) | 0 | 4360 (15.1) | 224 (17.1) | 0.046 |  |
|  | 1 | 24591 (84.9) | 1084 (82.9) |  |  |
