# Supplementary material for: Longitudinal trajectories of walking speed and risk of incident hip fracture in osteoporosis: a group-based trajectory modeling analysis from HRS, ELSA and SHARE
Source: Front Public Health. 2026 Jul 2;14:1857692. doi: 10.3389/fpubh.2026.1857692 (PMC13372962; doi:10.3389/fpubh.2026.1857692)
Supplement: Supplementary file 5 [file Supplementary_file_5.docx]

| Exposure Variable | HRS | | ELSA | | SHARE | |
| --- | --- | --- | --- | --- | --- | --- |
|  | HR (95% CI) | P-value | HR (95% CI) | P-value | HR (95% CI) | P-value |
| Log Walking speed | 1.06 (1.02 ~ 1.10) | 0.005** | 1.52 (1.01 ~ 2.28) | 0.046* | 1.03 (0.98 ~ 1.09) | 0.262 |
| Walking speed trajectory |  |  |  |  |  |  |
| Stable group | 1.00 (Reference) | - | 1.00 (Reference) | - | 1.00 (Reference) | - |
| Low−stable group | 1.79 (1.10 ~ 2.91) | 0.019* | 2.26 (1.48 ~ 3.45) | <.001*** | 1.02 (0.70 ~ 1.49) | 0.929 |
| Moderate−steep increasing group | 1.99 (1.31 ~ 3.03) | 0.001** | 2.96 (1.90 ~ 4.60) | <.001*** | 1.93 (1.24 ~ 3.02) | 0.004** |
| High−increasing group | 2.53 (1.48 ~ 4.35) | <.001*** | 3.67 (1.85 ~ 7.28) | <.001*** | 1.33 (0.64 ~ 2.75) | 0.448 |
| P for trend | 1.43 (1.22 ~ 1.68) | <.001*** | 1.46 (1.28 ~ 1.67) | <.001*** | 1.13 (1.03 ~ 1.23) | 0.010** |
| Adjust: Age, Sex, Race, Education, marital status, Wealth, BMI, Hypertension, Diabetes, Drinking status, Smoking status, Physical activity, Cardiovascular disease, Cancer | | | | | | |
| Significance: * p < 0.05, ** p < 0.01, *** p < 0.001 | | | | | | |

Supplementary Table 5. Sensitivity analyses of the associations between walking speed trajectories and incident hip fracture after additional adjustment for fall history in the HRS, ELSA, and SHARE cohorts
